# Biological Characteristics of Subsilicone Oil Fluid and Differences With Other Ocular Humors

**DOI:** 10.1167/tvst.8.1.28

**Published:** 2019-02-28

**Authors:** Hideyuki Shimizu, Hiroki Kaneko, Ayana Suzumura, Kei Takayama, Rina Namba, Yasuhito Funahashi, Keiko Kataoka, Takeshi Iwase, Shiang-Jyi Hwang, Seina Ito, Kazuhisa Yamada, Shinji Ueno, Yasuki Ito, Hiroko Terasaki

**Affiliations:** 1 Department of Ophthalmology, Nagoya University Graduate School of Medicine, Nagoya, Japan; 2 Department of Ophthalmology, National Defense Medical College, Nagoya, Japan; 3 Department of Urology, Nagoya University Graduate School of Medicine, Nagoya, Japan; 4 Laboratory of Bell Research Center–Department of Obstetrics and Gynecology Collaborative Research, Nagoya University Graduate School of Medicine, Nagoya, Japan

**Keywords:** subsilicone oil fluid, SORVL, cytokines

## Abstract

**Purpose:**

Subsilicone oil fluid (SOF) in eyes with silicone oil (SO) endotamponade possibly has a role in complications (e.g., vision loss); thus, we aimed to examine inflammatory cytokine and electrolyte levels and retinal glial cell viability in SOF.

**Methods:**

We measured major inflammatory cytokine levels and electrolytes in SOF and compared them with those in vitreous fluid (VF) and anterior chamber fluid (ACF). We analyzed the correlation between inflammatory cytokines and retinal thickness in SO-filled eyes. Further, we measured the MIO-M1 cell viability in medium with SOF and compared it with that containing VF.

**Results:**

We collected and examined 57 SOF, 22 ACF, and 21 VF samples from eyes with PVR, PDR, RD, and MH. Interleukin (IL)-8 and monocyte chemoattractant protein (MCP)-1 levels in SOF were significantly higher than those in ACF. There was no significant difference for all cytokines between SOF and VF. Retinal thickness changes during SO endotamponade were not correlated with the presence of any inflammatory cytokines. Levels of ferrous iron, but not of potassium, showed a significant decrease in SOF compared with VF. The WST-1 assay showed that SOF-added medium induced higher MIO-M1 cell viability than VF-added medium.

**Conclusions:**

We found no significant correlation between the change in the retinal thickness and cytokine levels, but SOF contains higher concentrations of cytokines and lower concentrations of ferrous iron and can be biologically distinguished from ACF and VF.

**Translational Relevance:**

Novel knowledge of inflammatory cytokine levels and electrolytes in SOF provides better understanding of pathology of SO-filled eyes.

## Introduction

Silicone oil (SO) is a major surgical adjuvant during retinal surgeries. It was first used in the 1960s; since then, its risks and benefits have been debated.^1^ SO-related vision loss (SORVL) cases have been reported, with unexplained vision loss during SO endotamponade and after SO removal.^2^–^4^ Understanding biological phenomena in SO-filled eyes is critical to improving SO indications during retinal surgery. SO, from a vitrectomy with SO tamponade, is evacuated after the retina has attached or the condition has stabilized, usually weeks to months after the primary surgery.^5^,^6^ During the evacuation, a certain amount of fluid in the space between SO and the surface of the posterior retina can be found in the eye. We have dubbed this fluid “subsilicone oil fluid (SOF)” and have hypothesized that inflammatory cytokines in it have pivotal roles in SORVL induction.^7^ We have proposed a safe method to extract this SOF and have examined the levels of major inflammatory cytokines in the SOF from eyes with proliferative vitreoretinopathy (PVR), proliferative diabetic retinopathy (PDR), retinal detachment (RD), and macular hole (MH)-associated retinal detachment. In that report, we found disease-specific cytokine profiles in SOF associated with disease status. However, we could not find specific causes for SORVL in SO-filled eyes. We designed this new study seeking to explain SORVL and SO-related retinal changes biologically, and we further examined the association between retinal thickness and cytokine level changes, and the cytokine level differences among SOF, anterior chamber fluid (ACF), and vitreous fluid (VF) in SO-filled eyes with PVR, PDR, or RD. We also assessed the differences in major electrolyte levels between SOF and VF. Finally, we examined culture retinal glial cell viability changes affected by the presence of SOF in vitro.

## Methods

### Sample Collection and Patient Diseases

For this study, we collected SOF, ACF, and VF samples from the eyes of patients with RD, PDR, PVR, and MH. We conducted the study by adhering to the guidelines of the Declaration of Helsinki, and the Nagoya University Hospital Ethics Review Board approved the protocol. We obtained written informed consent from all participating patients. We collected SOF samples as described.^7^ Briefly, before the beginning of the infusion, the edge of a 25-G blunt needle was placed above the surface of the posterior retina in a SO-filled eye using a RESIGHT surgical microscope (Zeiss, Oberkochen, Germany). Next, surgeons aspirated the SOF while monitoring the fundus. We collected all VF samples by dry vitrectomy at the beginning of the vitrectomy surgeries using a vitrectomy cutter before initiating infusion. Finally, we collected ACF samples from eyes that had migrated SO microbubbles in the anterior chamber at the time of washing out the microbubbles. All samples were centrifuged, and we used only the supernatants. We stored the samples at −80°C until use.

### Retinal Thickness Measurement

We measured retinal thicknesses (average retinal thickness within 1000-μm diameters centered from the fovea and 4 sectors around the fovea) according to the early treatment diabetic retinopathy study (ETDRS) chart,^8^ 1 month after the first vitrectomy surgery and just before the SO evacuation surgery. We analyzed possible associations between the retinal thickness changes and SOF cytokine levels.

### Measurement of Inflammatory Cytokines and Electrolytes

We froze SOF, ACF, and VF and thawed them only once before applying the MILLIPLEX MAP Human Cytokine/Chemokine Panel (Merck Millipore, Billerica, MA), a bead-based multiplex immunoassay that allows the simultaneous quantification of the following human cytokines: fibroblast growth factor (FGF)-2, interferon (IFN)-γ, interleukin (IL)-10, IL-12p40, IL-1β, IL-6, IL-8, monocyte chemoattractant protein (MCP)-1, tumor necrosis factor (TNF)-α, and vascular endothelial growth factor (VEGF). We used values of “0” for samples under the detection sensitivity in the statistical analyses. We also measured electrolytes (Na, K, Cl, Ca, Fe, Mg, and Zn) using a LABOSPECT 008 (Hitachi High-Technologies Corporation, Tokyo, Japan) in SOF and VF and compared the average values.

### Measurement of MIO-M1 Cell Viability Exposed to SOF and VF

We performed experiments to examine the biological effect of SOF in human Müller cells. The cultured MIO-M1 cells, purchased from E-lucid (University College London, London, UK) and cultured in Dulbecco's modified Eagle's medium (Thermo Fisher Scientific, Waltham, MA) with 10% fetal bovine serum (Gibco, Waltham, MA) and 1% penicillin–streptomycin (Merck KGaA, Darmstadt, Germany), were placed in medium supplemented with 50% (vol/vol) SOF from patients with PVR, PDR, or RD or supplemented with VF from patients with MH as control. After 4-hour incubations, we measured the cell viability of each sample using Cell Proliferation Reagent WST-1 (Merck KGaA, Darmstadt, Germany).

### Statistics

We expressed data as means ± standard error (SE; *n* = number of samples). In cases where one patient received treatment for both the right and left eyes, we counted each eye individually (*n* = 2). We compared the cytokine levels in several groups using the Kruskal–Wallis test and applied a Scheffe test in cases with significant differences (*P* < 0.05). We analyzed the possible correlation between retinal thickness and cytokine levels using Spearman's rank correlation. *P* values less than 0.05 were considered statistically significant in all analysis.

## Results

### Patients' Characteristics

In total, we collected 57 SOF, 22 ACF, and 21 VF samples for this study. All VF samples were collected during the vitrectomy surgery. The primary retinal diseases were RD, PVR, and PDR, and Table 1 lists the patients' characteristics. Of the 57 SOF samples, the cytokine levels of 55 samples were used to evaluate their association with the retinal thickness; ACF was extracted from 22 eyes; VF was extracted from 11 eyes at the time of primary vitrectomy surgeries; and 10 SOF samples were used for electrolyte measurement.

**Table 1:** Patients' Characteristics

*N* of Patients (Male)	Age	Duration of SO Tamponade, mo
RD		
20 (14)	56.1 ± 18.9	4.7 ± 3.1
PVR		
14 (10)	50.6 ± 25.3	4.0 ± 2.1
PDR		
23 (13)	46.9 ± 10.8	4.4 ± 2.1

### Cytokine Levels in SOF and ACF

**Figure 1:**
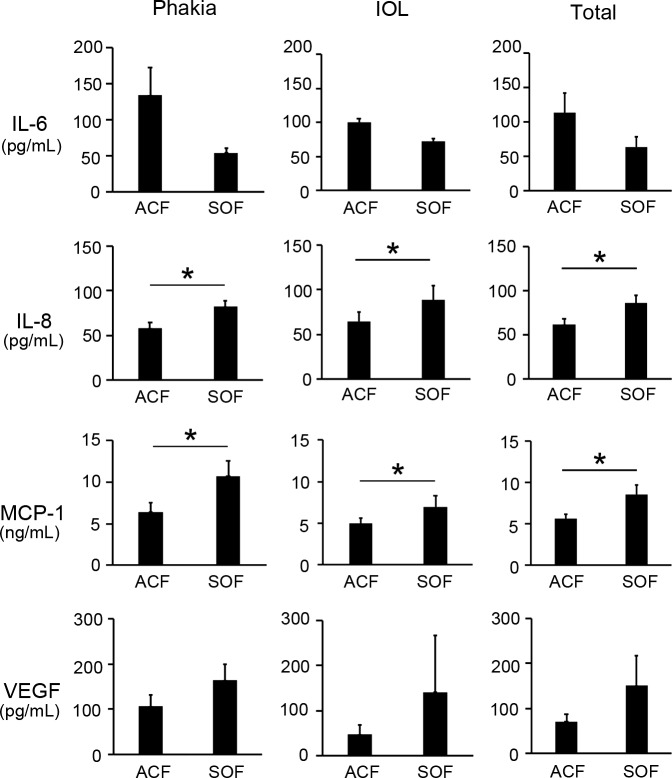
Cytokine levels in SOF and ACF. Out of the major inflammatory cytokines, IL-6, IL-8, MCP-1, and VEGF were measured from all samples. IL-8 and MCP-1 were significantly higher in SOF than in ACF. These characteristics were preserved even after dividing all ACF samples (n = 22) into a group of eyes with clear lens (phakia, n = 13) and a group of eyes with IOL implantation (n = 9). *P < 0.05.

The major inflammatory cytokine levels in SOF (*n* = 22) and ACF (*n* = 22) from the same eyes (*n* = 22) are listed in Table 2. We did not detect FGF-2, IFN-γ, IL-1β, or TNF-α in all samples (“0”). Figure 1 shows the comparisons of cytokine levels (IL-6, IL-8, MCP-1, and VEGF) between SOF and ACF from the same eyes. IL-8 expression in SOF was 85.72 ± 8.94 pg/mL and significantly higher (1.4-fold) than in ACF (61.89 ± 5.89 pg/mL). Moreover, MCP-1 expression in SOF (8526 ± 1092 pg/mL) was significantly higher (1.5-fold) than that in ACF (5559 ± 607 pg/mL). However, we found no significant differences in the other cytokines between SOF and ACF. We further analyzed the differences in the cytokine levels of IL-6, IL-8, MCP-1, and VEGF between SOF and ACF by dividing all 22 ACF samples into two groups as follows: eyes with clear lens (phakia, *n* = 9), and eyes with intraocular lens implantation histories (IOL, *n* = 13). The mean IL-8 levels in SOF were significantly higher than those in the ACF_phakia (1.4-fold) and ACF_IOL (1.5-fold) samples. Similarly, the mean MCP-1 levels in SOF were significantly higher than those in the ACF_phakia (1.4-fold) and ACF_IOL (1.7-fold) samples. However, we found no significant differences in the other cytokines between the SOF and ACF samples even after dividing into ACF_phakia and ACF_IOL groups.

**Table 2:** Cytokine Levels in ACF and SOF

	FGF-2, pg/mL	IL-10, pg/mL	IFN-γ, pg/mL	IL-12p40, pg/mL	IL-6, pg/mL
ACF	0 ± 0	2.91 ± 1.67	0 ± 0	0.99 ± 0.99	114.0 ± 28.71
SOF	0 ± 0	3.20 ± 1.59	0 ± 0	0.99 ± 0.99	64.35 ± 14.05

**Table 2:** Extended

	IL-8, pg/mL	MCP-1, pg/mL	TNF-α, pg/mL	VEGF, pg/mL	IL-1β, pg/mL
ACF	61.89 ± 5.89	5559 ± 607	0 ± 0	70.86 ± 16.97	0 ± 0
SOF	85.72 ± 8.94	8526 ± 1092	0 ± 0	150.4 ± 67.56	0 ± 0

### Cytokine Levels of the SOF and VF Samples

The major inflammatory cytokines of the SOF (*n* = 11) and VF (*n* = 11) samples from the same eyes (*n* = 11) are listed in Table 3. We did not detect IFN-γ, IL-1β, or TNF-α in all samples (“0”), and we found no significant differences in all cytokines between these two fluid types.

**Table 3:** Cytokine Levels in VF and SOF

	FGF-2, pg/mL	IL-10, pg/mL	IFN-γ, pg/mL	IL-12p40, pg/mL	IL-6, pg/mL
VF	16.97 ± 5.17	3.62 ± 0.93	0 ± 0	2.83 ± 0.89	99.12 ± 19.54
SOF	38.52 ± 14.92	4.20 ± 1.27	0 ± 0	4.85 ± 1.37	66.57 ± 23.64

**Table 3:** Extended

	IL-8, pg/mL	MCP-1, pg/mL	TNF-α, pg/mL	VEGF, pg/mL	IL-1β, pg/mL
VF	85.90 ± 16.22	6804 ± 809	0 ± 0	71.75 ± 22.69	0 ± 0
SOF	80.54 ± 33.43	6183 ± 870	0 ± 0	51.49 ± 10.44	0 ± 0

### Association Between Retinal Thickness Changes and Cytokine Levels in SOF

The average retinal thickness changes in each sector from eyes with PVR, PDR, and RD are listed in Table 4. In eyes with PDR and RD, the foveal and average (of 5 ETDRS sectors) retinal thicknesses were higher at the time of SO evacuation than those 1 month after SO injection. In contrast, the foveal and average retinal thicknesses in PVR were lower at the time of SO evacuation than those 1 month after SO injection. We found no significant differences in the retinal thickness changes among all groups (PVR, PDR, and RD). In eyes with PVR, PDR, and RD, we found significant correlations between the foveal retinal thickness changes and the average retinal thickness (*r* = 0.66, 0.94, and 0.69; *P* < 0.05, 0.01, and 0.01, respectively). In eyes with PDR, the duration of SO endotamponade was positively correlated with the IL-6 (*r* = 0.66, *P* < 0.05) and IL-8 (*r* = −0.45, *P* < 0.01) in SOF samples. However, we found no significant correlations between the retinal thickness change and either of the inflammatory cytokine levels (Table 5).

**Table 4:** Average Change of the Retinal Thickness in Eyes

	Superior Inner Macula, μm	Inferior Inner Macula, μm	Fovea, μm	Temporal Inner Macula, μm	Nasal Inner Macula, μm	Total Change of 5 Sectors, μm	Average Change, μm
RD	3.89 ± 9.49	16.68 ± 10.60	9.63 ± 14.90	6.37 ± 10.21	15.84 ± 10.40	52.42 ± 42.84	10.48 ± 8.57
PVR	−19.21 ± 14.80	2.5 ± 10.44	−38.21 ± 15.75	−11.85 ± 13.52	6.21 ± 15.80	−60.57 ± 50.24	−12.11 ± 10.05
PDR	20.72 ± 29.20	48 ± 22.80	26.95 ± 24.15	18.18 ± 23.16	34.36 ± 29.33	148.2 ± 116.9	29.64 ± 23.37

**Table 5:** Correlation Between the Changes of Retinal Thickness and Cytokine Levels in SOF

	RD			PVR			PDR		
	Average Change in Retinal Thickness	Change in Foveal Thickness	Duration of SO Tamponade	Average Change in Retinal Thickness	Change in Foveal Thickness	Duration of SO Tamponade	Average Change in Retinal Thickness	Change in Foveal Thickness	Duration of SO Tamponade
Average change in retinal thickness	-			-			-		
Change in foveal thickness	0.69*	-		0.66*	-		0.94*	-	
Duration of SO tamponade	0.19	0.17	-	0.35	−0.2	-	0.33	0.39	-
IL-6	0.31	0.04	0.05	0.08	−0.24	0.31	0.30	0.31	0.66*
IL-8	0.41	0.41	0.04	0.02	−0.07	−0.03	−0.11	−0.18	−0.45*
MCP-1	0.26	0.16	−0.15	0.18	−0.16	0.29	−0.27	−0.29	−0.31
TNFα	0.15	0.08	0.37	0.04	0.06	−0.30	0.18	0.14	−0.35
VEGF	−0.04	−0.32	0.21	0.24	0.35	0.34	−0.40	−0.42	−0.14

* *P* < 0.05.

### Electrolyte Levels in SOF and VF

**Figure 2:**
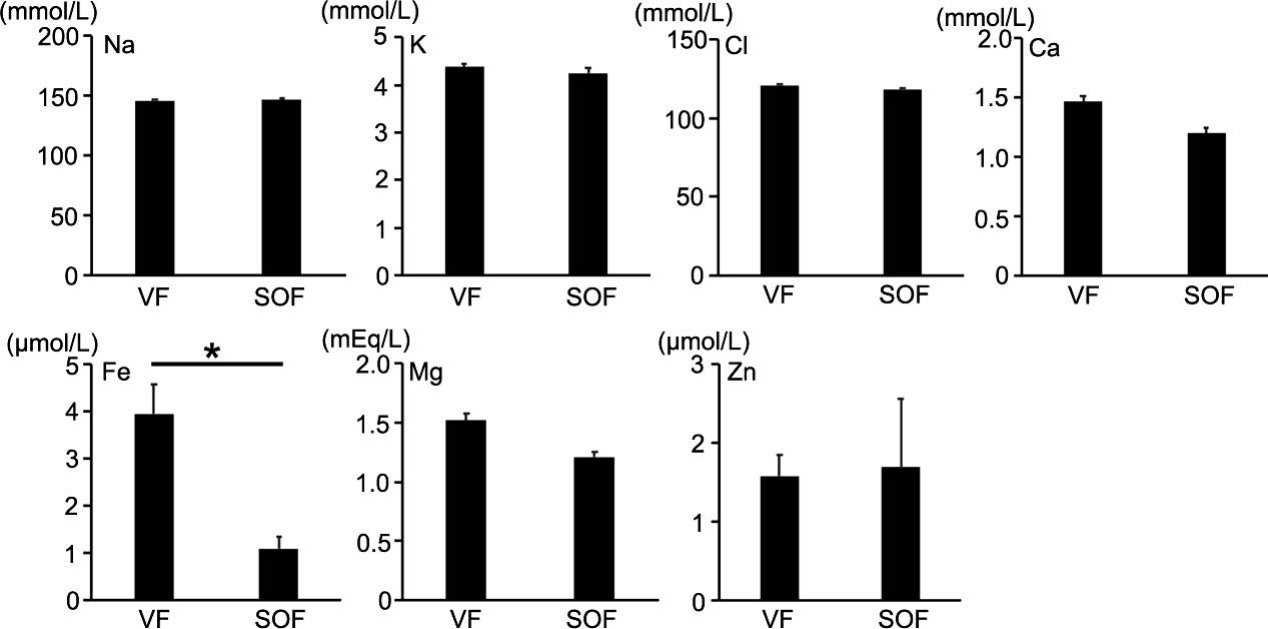
Electrolyte levels in SOF and VF. When comparing the electrolyte levels in VF and SOF, only ferrous iron (Fe) showed a significant difference. *P < 0.05. VF, vitreous fluid.

We measured electrolytes in 10 SOF and 10 VF samples and compared them. The ferrous iron concentrations were 1.07 mmol/mL in SOF and 4.00 mmol/mL in VF, and the difference was statistically significant (*P* < 0.001). The Na, K, Cl, Ca, Mg, and Zn concentrations were 146.90, 4.23, 117.80, 1.21, 1.21, and 1.70 mmol/mL in SOF and 145.90, 4.38, 120.60, 1.47, 1.52, and 1.58 mmol/mL in VF. Thus, we found no significant differences between the SOF and VF levels (Fig. 2).

### MIO-M1 Cell Viability Exposed to SOF and VF

**Figure 3:**
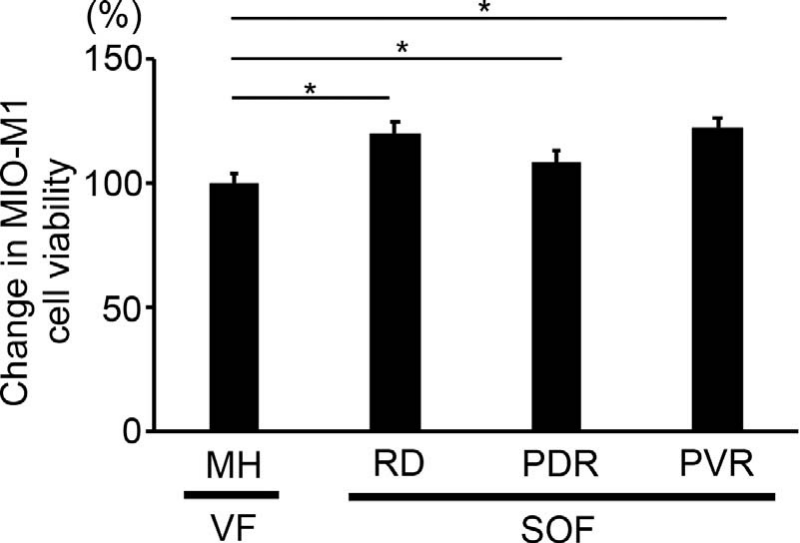
MIO-M1 cell viability under different culture conditions. The cell viabilities of human Müller cells (MIO-M1 cells) were tested in media containing either SOF from eyes with PVR, PDR, and RD or VF from eyes with MH. The cells exposed to SOF had higher viabilities (120%, 109%, and 122%, respectively) than those exposed to VF (control, 100%). *P < 0.05.

We analyzed the viability of MIO-M1 cells cultured in a medium containing 50% (vol/vol) SOF from eyes with PVR, PDR, and RD and compared them with those in medium containing 50% (vol/vol) VF from eyes with MH. Compared with the cell viability in MH_VF (control, 100%), those in PVR, PDR, and RD SOFs were significantly higher at 123%, 109%, and 120%, respectively (*P* = 0.033, 0.013, and 0.009, respectively; Fig. 3).

## Discussion

Although SO has been frequently used, SORVL has emerged as a newly recognized phenomenon causing visual impairment for unexplained reasons. We used modern surgical machines and devices to safely collect SOF samples and examined their characteristics in detail. On the basis of the hypothesis that SOF has an important role in the pathogenesis of SORVL, we analyzed the inflammatory cytokine and electrolyte contents of SOF samples. We had already reported our finding that IL-6 and TNF-α levels in SOF samples were higher in eyes with revision surgery required–PVR than in those with simple PVR needing only SO evacuations at the time of the second surgeries.^7^ In contrast, FGF-2, IL-10, IL-12p40, IL-8, VEGF, and TGF-β1 were higher in the SOFs of eyes with revision surgery required–PDR than in those needing simple PDR.^7^ While examining the SOF contents, we wondered whether differences in inflammatory cytokine contents existed between SOF and ACF. We found that the IL-8 and MCP-1 levels in SOF were significantly higher than those in ACF, suggesting that SOF and ACF are not identical in the eyes. Additionally, we explored possible differences in SOF inflammatory cytokine levels, depending on eye lens differences (phakic eyes or IOL-implanted eyes). Our results indicate that the lens status is independent of the difference between SOF and ACF cytokine contents. Between SOF and ACF, we observed a difference in IL-8 and MCP-1 after the inclusion of the eyes with RD, PDR, and PVR in one group. We found a similar difference between SOF and ACF even when RD, PDR, and PVR were separately examined (Supplementary Fig. S1). On the other hand, there were no significant differences in major cytokine levels between VF and SOF obtained from the same eyes. Cytokine levels in VF at the time of primary vitrectomy surgeries might vary depending on the severity of diseases and timing of the surgeries. For instance, in cases with PDR, histories of anti-VEGF treatment, or laser photocoagulation might affect the results.^9^–^11^

Retinal thickness is increased in cases with diabetic macular edema or cystoid macular edema.^12^,^13^ In contrast, retinal thinning could become a problem in retinal diseases.^14^,^15^ Retinal thickness may reflect the retinal disease status. Therefore, we examined the correlation between retinal thickness and inflammatory cytokine levels in samples from SO-filled eyes. Interestingly, the retinal thicknesses in eyes with PVR undergoing SO endotamponade were thinner than those in healthy eyes, but they were thicker in eyes with PDR and RD undergoing SO endotamponade. We previously reported, in cases with PDR undergoing SO endotamponade, the retinal thickness decreased after SO evacuation.^8^ In this study, however, we found that the retinal thickness increased in eyes with PDR that were filled with SO. Corroboration of these data suggested that, in cases of PDR that involved SO endotamponade, retinal thickness increased when SO was being filled in the eyes but decreased after SO was evacuated. In addition, our current study indicated that the retinal thickness decreased in eyes with PVR while SO was being filled in the eyes. On the basis of the difference in SOF cytokines between eyes with PVR and eyes with PDR, specific factor differences may affect retinal thicknesses in SO-filled eyes. We further hypothesized that some cytokine levels are correlated with the retinal thickness in SO-filled eyes. However, in this study, we did not find significant correlations of any cytokines with retinal thickness change. Studies have shown major inflammatory cytokine increases in the VF of eyes with PDR.^16^–^19^ This indicates a pivotal role of VEGF in the VF of PDR.^20^–^24^ The VEGF SOF levels that we measured ranged from 0 to 296 pg/mL. In contrast, the same levels in eyes with PDR were reported to range from 585.7 to 1316.2 pg/mL^20^,^25^; thus, the VEGF levels in SOF are lower than those in VF. This suggests that the VEGF SOF level either is not involved in retinal thickness in PDR or plays a smaller role than the VEGF VF level. We found a positive correlation between the duration of SO endotamponade and the IL-6 SOF level in eyes with PDR, and a negative correlation between the duration of SO endotamponade and the IL-8 SOF level. IL-6 is one of the major proinflammatory cytokines with neuroprotective roles for photoreceptors.^26^,^27^ IL-8 plays an important role in ocular inflammation and angiogenesis.^28^ PDR is considered a chronic inflammatory condition,^29^–^36^ and exposure to these proinflammatory cytokines for months may affect the retinal thickness changes in SO-filled eyes with PDR.

At the end of the vitreous side of the retina, the internal limiting membrane (ILM) is present. ILM is believed to be the basal membrane of Müller cells, and ILM (or Müller cells) could be the cells that highly exposed to SOF in the eye. Therefore, we decided to examine Müller cell (MIO-M1 cells) viability after the exposure to SOF in vitro. In our investigation on the viability/proliferative activity of MIO-M1 cells exposed to SOF, we found higher cell viability in MIO-M1 cells exposed to SOF than in those exposed to control VF. MH is caused by posterior vitreous membrane traction and is believed to not be associated with inflammation.^37^–^39^ Therefore, VF from eyes with MH was used as a control. Our results suggest that cytokine-related factors in SOF affect Müller cell viability in SO-filled eyes. Recent studies revealed that Müller cells are important as they secrete neurotrophic factors.^40^–^44^ Although possibly not directly related to the pathogenesis of SORVL, the finding that SOF increased Müller cell viability could be important to understand the biological changes in SO-filled eyes. On the other hand, it is highly possible that the retinal cells other than Müller cell (e.g., retinal ganglion cells [RGC]), are also affected by SOF. For instance, in eyes with glaucoma, RGC loss is strongly associated with the severity of glaucoma, whereas high prevalence of glaucoma has been reported in SO-filled eyes.^45^–^47^ Therefore, estimation of cell viability of RGC in addition to that of Müller cell is beneficial in understanding SORVL.^48^–^50^

Scheerlinck et al.^51^ examined the electrolyte levels in SOF after RD surgeries and found that magnesium ions (Mg) and chloride ions (Cl) were significantly lower than those in VF. However, in our study, we found no significant differences in Mg and Cl between the SOF and VF samples. In contrast, we found that the ferrous iron levels were significantly lower in SOF than in VF. The discrepancy in the results between the previous report^51^ and our current study may be caused by the difference in the diversity of SOF samples. In the former study,^51^ SOF samples were collected only from eyes with RD, and control VF samples were collected from eyes with MH or floaters. In contrast, we used SOF from eyes not only with RD but also with PVR or PDR. Previous studies suggest that potassium (K) might cause retinal toxicity.^52^ Interestingly, the K levels were not high in SOF and did not show significant differences when comparing them with control VF both in our current study and the previous study.^51^

The limitations of this study include the following: (1) all SOF and VF samples were not collected from the same eyes. As we previously reported,^1^ the cytokine levels in samples from eyes with PVR and PDR varied. Thus, collecting cytokine and electrolyte data from the same eye would enable us to obtain more precise information. (2) We did not analyze data from different disease types (PVR, PDR, RD, and MH) separately in our cytokine analysis of ACF, VF, and SOF. As shown in Supplementary Figure S1, we obtained similar results regarding the difference between SOF and ACF when different disease types were separately analyzed. However, further analysis with increased number of samples could provide additional findings. (3) Although it is desirable to use samples from MH, a typical noninflammatory disease, as a control, we have rarely encountered cases with MH requiring SO endotamponade; thus, retrieving SOF samples from eyes with MH is difficult.^53^–^55^ (4) Moreover, because the collected SOF amounts were very small, it was difficult to measure cytokine levels and electrolytes from the same fluid samples. (5) For the same reason, we could not measure MIO-M1 cell viability at different doses (vol/vol, %) to obtain dose-dependent cell viability differences.

We were not able to find specific reasons for SORVL in our study, but we obtained important information on SOF by measuring the cytokine and electrolyte contents and by comparing the values among different eye fluids. Current surgical devices allow retinal surgeons to obtain SOF samples easily and safely, and future studies on SOF are needed to increase our understanding of SORVL.

## Supplementary Material

Supplement 1
