# Supplementary material for: Biological Characteristics of Subsilicone Oil Fluid and Differences With Other Ocular Humors
Source: Transl Vis Sci Technol. 2019 Feb 28;8(1):28. doi: 10.1167/tvst.8.1.28 (PMC6398349; doi:10.1167/tvst.8.1.28)
Supplement: Supplement 1 [file tvst-08-01-14_s01.pdf]

Supplementary Figure

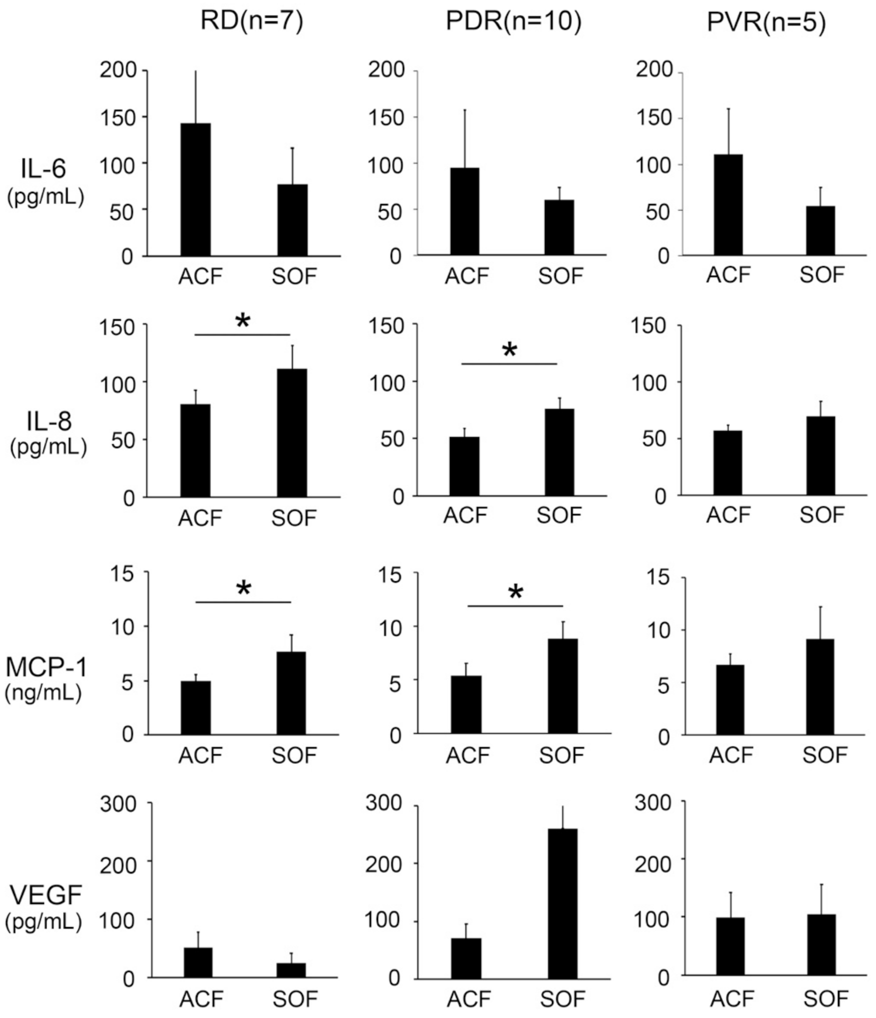

Major inflammatory cytokines, interleukin (IL)-6, IL-8, monocyte chemoattractant protein (MCP)-1, and vascular endothelial growth factor (VEGF) in sub-silicone oil fluid (SOF) and anterior chamber fluid (ACF) were measured from eyes with retinal detachment (RD, n=7), proliferative diabetic retinopathy (PDR, n=10), and proliferative vitreoretinopathy (PVR, n=5). IL-8 and MCP-1 were significantly higher in SOF than in ACF in eyes with RD and PDR. $^{*}P < 0.05$.
